# Efficacy and Safety of Combination Therapy with Low-Dose Rivaroxaban in Patients with Cardiovascular Disease: A Systematic Review and Meta-Analysis of Randomized Controlled Trials

**DOI:** 10.3390/jcm13072033

**Published:** 2024-03-31

**Authors:** Tommaso Bucci, Francesco Del Sole, Danilo Menichelli, Gioacchino Galardo, Flavio Giuseppe Biccirè, Alessio Farcomeni, Gregory Y. H. Lip, Pasquale Pignatelli, Daniele Pastori

**Affiliations:** 1 Liverpool Centre for Cardiovascular Science at University of Liverpool, Liverpool John Moores University and Liverpool and Heart and Chest Hospital, Liverpool L7 8TX, UKlipgy@liverpool.ac.uk (G.Y.H.L.); daniele.pastori@uniroma1.it (D.P.); 2 Department of General and Specialized Surgery, Sapienza University of Rome, 00161 Rome, Italyg.galardo@policlinicoumberto1.it (G.G.); flaviogiuseppe.biccire@uniroma1.it (F.G.B.); 3 Department of Clinical, Internal Medicine, Anaesthesiological and Cardiovascular Sciences, Sapienza University of Rome, 00161 Rome, Italy; francesco.delsole@uniroma1.it; 4 Department of Economics and Finance, University of Rome “Tor Vergata,” Via Columbia 2, 00133 Rome, Italy; 5 Danish Center for Health Services Research, Department of Clinical Medicine, Aalborg University, 9220 Aalborg, Denmark

**Keywords:** rivaroxaban, coronary artery disease, peripheral artery disease, bleeding, major adverse cardiovascular events

## Abstract

**Objectives**: To review the evidence on the effectiveness and safety of low-dose-rivaroxaban 2.5 mg twice daily (LDR) in patients with coronary artery disease (CAD) and/or peripheral artery disease (PAD) taking antiplatelets. **Methods**: We performed a systematic review and meta-analysis of randomized controlled trials (RCTs). Efficacy endpoints were cardiovascular events (CVEs), myocardial infarction, stroke, all-cause, and cardiovascular death. Any, major, fatal bleeding, and intracranial hemorrhage (ICH) were safety endpoints. Numbers needed to treat (NNT), and numbers needed to harm (NNH) were also calculated. **Results**: Seven RCTs were included with 45,836 patients: 34,276 with CAD and 11,560 with PAD. Overall, 4247 CVEs and 3082 bleedings were registered. LDR in association with either any antiplatelet drug or aspirin (ASA) alone reduced the risk of CVEs (hazard ratio [HR] 0.86, 95% confidence interval [95%CI] 0.78–0.94) and ischemic stroke (HR 0.68, 95%CI 0.55–0.84). LDR + ASA increased the risk of major bleeding (HR 1.71, 95%CI 1.38–2.11) but no excess of fatal bleeding or ICH was found. The NNT to prevent one CVE for LDR + ASA was 63 (43–103) and the NNH to cause major bleeding was 107 (77–193). **Conclusions**: The combination of LDR with either antiplatelet drugs or low-dose aspirin reduces CVEs and ischemic stroke in patients with CAD/PAD. There was an increased risk of major bleeding but no excess of fatal or ICH was found. LDR seems to have a favorable net clinical benefit compared to ASA treatment alone.

## 1. Introduction

Coronary artery disease (CAD) and peripheral artery disease (PAD) are two clinical conditions that often coexist, sharing the atherosclerotic process as a common pathophysiological mechanism. Nearly 40% of patients diagnosed with CAD concurrently have PAD [1], while approximately 50% of those diagnosed with PAD also have CAD [2]. This dual occurrence significantly amplifies the risk of adverse cardiovascular events (CVEs) when compared to individuals afflicted with either condition alone [3]. Indeed, patients with CAD and PAD have a cumulative risk for recurrent CVEs of 25% after 3 years and 17.6% after 4 years, despite recommended secondary prevention strategies [4,5,6]. Antiplatelet therapy, either single or double, along with statins, remains the cornerstone of the secondary prevention strategy in these high-risk patients [7,8]. However, the high rate of recurrent cardiovascular events in patients with PAD and/or CAD suggests that antiplatelet drugs, either aspirin (ASA) or P2Y12-inhibitor (or a combination of them), are not sufficient to reduce the risk of recurrent thrombotic events in these patients [9]. Starting from this evidence, several studies investigated the effect of combination therapies by the addition of vitamin K antagonists or direct oral anticoagulants to antiplatelet drugs on residual cardiovascular risk [10,11,12,13,14,15]. The physiopathology behind this therapeutic approach relies on the fact that platelets and the coagulation system can crosstalk in different ways. Indeed, thrombin can directly activate the platelet by binding the protease-activated receptor (PAR-1), and the release of factor V from the platelet’s granules contributes to the formation of the prothrombinase complex with factor X at the site of plaque rupture [16]. Previous in vitro and in vivo evidence showed that the anti Xa oral anticoagulant can interrupt this cross-talk blocking both the thrombin generation and the platelet activation through the glycoprotein VI shedding [17,18] and the inhibition of PAR-1 [19].

Such dual pathway inhibition has been tested in randomized controlled trials, such as the COMPASS (Cardiovascular Outcomes for People Using Anticoagulation Strategies) study that included patients with either CAD or PAD or both and the VOYAGER (Vascular Outcomes study of ASA along with rivaroxaban in endovascular or surgical limb revascularization for peripheral artery disease) study in patients with PAD. These studies showed that low-dose rivaroxaban 2.5 mg bid (LDR) plus ASA reduced the rate of cardiovascular events compared to the antiplatelet regimen alone, despite an increase in the risk of major bleeding [20,21]. Based on these results, the European Medicines Agency has approved LDR for the prevention of recurrent adverse CVEs in patients with CAD and PAD and the European Cardiology Society (ESC) indicates the dual antithrombotic therapy with LDR with ASA as a possible option for the long-term antithrombotic treatment in patients with CAD at high risk [22]. However, no clear indication has been provided from the main European and North American guidelines for PAD management regarding the use of LDR in this clinical context [7,23]. Moreover, the net clinical benefit between thrombotic risk reduction and increased risk of bleeding of this dual antithrombotic approach in patients at high risk of CVEs is not completely understood.

Two previous meta-analyses included acute coronary syndrome patients only [24,25], whereas another meta-analysis included different DOACs, making the generalizability of their findings difficult [26].

For this reason, we conducted a systematic review and meta-analysis of the randomized controlled trials with at least one treatment arm containing LDR and an antiplatelet drug to evaluate the following: (1) efficacy endpoints: risk of cardiovascular events, MI, stroke, cardiovascular death, and all-cause mortality; (2) safety endpoints: any and major bleeding, intracranial hemorrhage and fatal bleeding; (3) the net benefit of LDR plus ASA compared to ASA alone, which is the guideline recommended therapeutic regimen.

## 2. Methods

### 2.1. Searches Strategy and Study Selection

**Figure 1:**
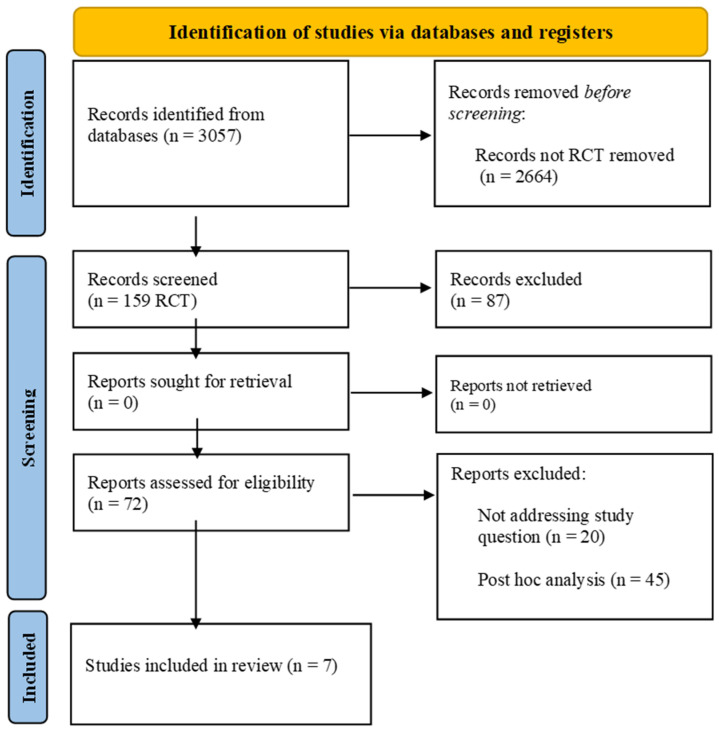
PRISMA flow diagram.

From 1 December 2021 to 31 December 2023, we researched MEDLINE (PubMed), Embase, the Cumulative Index to Nursing and Allied Health Literature, the Cochrane Central Register of Controlled Trials in the Cochrane Library, and the WHO Global Index Medicus for potentially relevant results. The search strategy included “rivaroxaban”, “coronary artery disease”, and “peripheral artery disease” as keywords and is detailed in Supplementary Table S1. The search strategy was performed according to PRISMA guidelines Figure 1. The initial inclusion criteria were as follows: (1) English language, (2) full-text articles available, (3) randomized controlled trials (RCTs), (4) the study condition was the presence of PAD or CAD (older than 18 years). Case reports/case series, observational studies, as well as reviews or editorials/letters were excluded. Retrieved citations were screened by title and abstract independently. Full texts of potentially relevant citations were assessed for the final decision of inclusion or exclusion, and disagreements were solved by collegial discussion. This study is registered as PROSPERO n°CRD42024518240.

### 2.2. Data Extraction

From the included studies, we collected data on author name, year of publication, study design, mean age, proportion of women/men, total patients, treatment, and control arms with administered dose. All studies and outcome data were collected in an electronic spreadsheet (Microsoft Excel, Office 365).

### 2.3. Risk-of-Bias Assessment

Two pairs of investigators (D.M. and T.B. and F.d.S. and G.G.) independently assessed the risk of bias (RoB) using the Cochrane RoB 2 tool for RCTs, that evaluates the following domains: randomization process, deviation from intended interventions, missing outcome data, measurement of the outcome, and selection of the reported result [27]. RoB 2 figures were created with the obvious online tool [28]. Publication bias was assessed with the realization of funnel plots (Supplementary Figures S1 and S2).

### 2.4. Treatment Groups

To obtain homogenous groups with a similar type of intervention, studies were divided into two groups: (1) LDR + aspirin (ASA) and/or P2Y12 inhibitor vs. ASA and/or P2Y12 inhibitor (Panel A in all the figures), and (2) LDR + ASA vs ASA (Panel B in all the figures).

### 2.5. Study Outcomes

Efficacy endpoints were a risk of CVEs, MI, stroke, cardiovascular death, and all-cause mortality. Safety endpoints were a risk of any bleeding, major bleeding, intracranial hemorrhage, and fatal bleeding. The definitions used for major bleeding and CVEs are reported in Table 1, while the definition of any bleeding and the number of events for each endpoint are reported in Table 2 and Table 3.

### 2.6. Statistical Analyses

A primary analysis was performed on all included studies regardless of the type of antiplatelet drug associated with LDR. We also performed a subgroup analysis including only studies with ASA as antiplatelet treatment. Moreover, given the wide heterogeneity of the population considered in this study, we performed two sensitivity analyses. These analyses focused separately on patients with CAD and those with PAD. In these analyses, instead of considering the original COMPASS study [12], we incorporated two different post hoc analyses that specifically addressed the risk of adverse events in CAD [20] or PAD [31] patients.

When not reported, hazard ratios (HR) and their standard errors were calculated based on the number of subjects, number of events, and mean follow-up per group. Meta-analyses for each endpoint were separately performed based on Bayesian random effect models, using the logarithm of hazard ratios (HR) as outcome. The Bayesian approach has been used since the number of studies involved was low [32]. Numbers needed to treat (NNT) and numbers needed to harm (NNH) were also calculated for LDR+ASA vs. ASA treatment, and their standard errors were assessed through bootstrap.

Analyses were performed using the R software (R development Core Team, 2021) version 4.1.2.

## 3. Results

We included a total of 45,836 patients, 34,276 with CAD, and 11,560 with PAD (Table 1). The mean age ranged from 54 to 70 years. Most patients included in the RCTs were men (>70% in all studies). In a mean follow-up of 524 ± 445 days, 4247 CVEs were registered. For the PIONEER [11], ATLAS ACS-TIMI 46 [10], and ATLAS ACS 2–TIMI 51 [29] studies, only the LDR 2.5 mg arm was considered.

### 3.1. LDR + Any Antiplatelet vs. Any Antiplatelet

For this analysis, 4247 CVEs and 3082 major bleedings were included.

**Figure 2:**
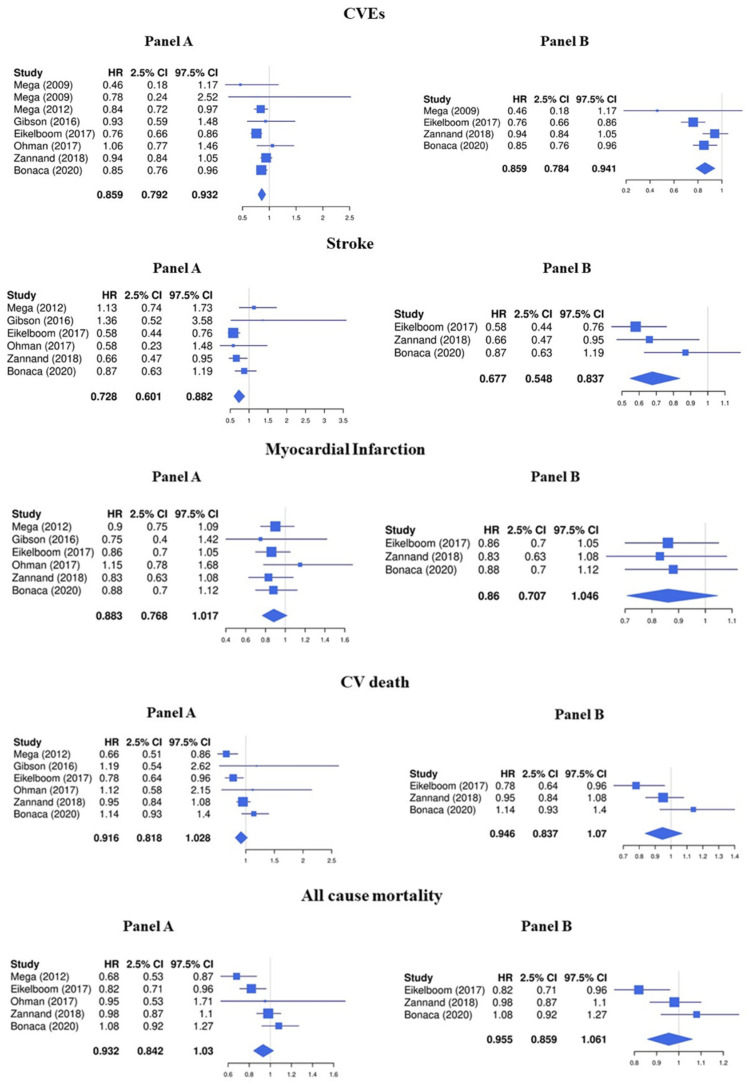
Efficacy endpoints (Panel **A**: LDR + any anti platelet vs. any anti platelet, Panel **B**: LDR + ASA vs. ASA only) [10,11,12,13,14,15,29].

The LDR + any antiplatelet treatment compared to regimens containing any antiplatelet drug alone significantly decreased the risk of CVEs (HR 0.86, 95%CI 0.79–0.93) (Figure 2, Panel A). In particular, LDR + any antiplatelet was associated with a significative lower risk of stroke (HR 0.73, 95%CI 0.60–0.88) while the protective effect against MI (HR 0.88, 95%CI 0.77–1.02), cardiovascular death (HR 0.93, 95%CI 0.84–1.03), and all-cause mortality (HR 0.92, 95%CI 0.82–1.03) was less pronounced (Figure 2, Panel A).

**Figure 3:**
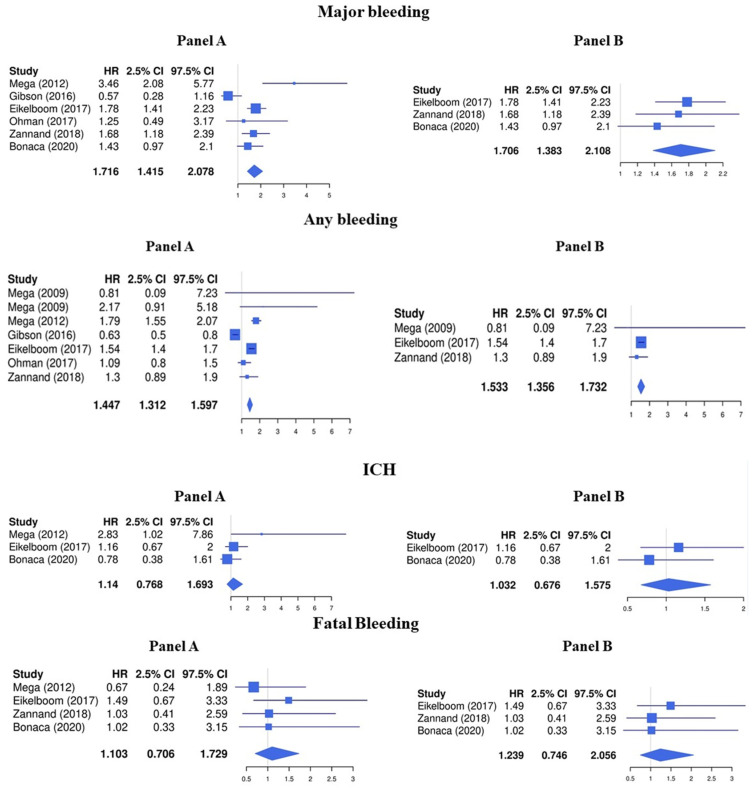
Safety endpoints (Panel **A**: LDR + any anti platelet vs. any anti platelet, Panel **B**: LDR + ASA vs. ASA only) [10,11,12,13,14,15,29].

Regarding the safety endpoints, LDR was associated with a higher risk of major bleeding (HR 1.72, 95%CI 1.42–2.08) and any bleeding (HR 1.45, 95%CI 1.31–1.60), while the risk for intracranial hemorrhage (HR 1.14, 95%CI 0.77–1.69) and fatal bleeding (HR 1.10, 95%CI 0.71–1.73) was not significantly increased (Figure 3, Panel A).

### 3.2. LDR + ASA vs. ASA Alone

We performed a subgroup analysis including 30,193 patients treated with LDR + ASA vs. ASA alone (Table 1). A total of 3290 CVEs and 1831 major bleeding were registered.

Consistent with the main analysis, the treatment with LDR + ASA reduced the risk of CVEs (HR 0.86, 95%CI 0.78–0.94) and stroke (HR 0.68, 95%CI 0.55–0.84) but was not associated with a concomitant statistically significant reduction of MI (HR 0.86, 95%CI 0.71–1.05), cardiovascular death (HR 0.96, 95%CI 0.86–1.06), and all-cause mortality (HR 0.95, 95%CI 0.84–1.07) (Figure 2, Panel B).

The risk of major bleeding (HR 1.71, 95%CI 1.38–2.11) and any bleeding (HR 1.53, 95%CI 1.36–1.73) was increased while no significative association was found with the risk of intracranial hemorrhage (HR 1.03, 95%CI 0.68–1.58) and fatal bleeding (HR 1.24, 95%CI 0.75–2.06) (Figure 3, Panel B).

The NNT and NNH for the LDR+ASA group showed an NNT to prevent one CVE of 63 (43–103), with a NNH of 107 (77–183).

### 3.3. Sensitivity Analyses

Analyzing only patients with CAD (Supplementary Figure S3), LDR was associated with a reduced risk of CVEs and stroke both when associated with any antiplatelets (HR 0.86, 95%CI 0.78–0.95, and HR 0.81, 0.65–1.00, respectively) or with ASA alone (HR 0.86, 95%CI 0.77–0.96 and HR 0.76, 95%CI 0.59–0.98, respectively). A reduced risk of CV death (HR 0.88, 95%CI 0.79–0.99) and MI (HR 0.87, 95%CI 0.77–0.99) was found when considering LDR associated with any antiplatelets, whereas the magnitude of this protective effect in those treated with ASA was less evident (HR for CV death 0.91, 95%CI 0.79–1.04 and HR for MI 0.86, 95%CI 0.73–1.01). Consistent with the main analysis, LDR was associated with a higher risk of any bleeding and major bleeding independently of the type of antiplatelet (Supplementary Figure S4), but not of intracranial hemorrhage or fatal bleeding (analysis performed only in patients treated with any antiplatelets).

All patients with PAD considered on the sensitivity analysis were on ASA. In this context, LDR was associated with a reduced risk of CVEs (HR 0.82, 95%CI 0.72–0.94), and a higher risk of major bleeding (HR 1.53, 95%CI 1.11–2.10). Non-significant trends for a protective effect of LDR were found for stroke (HR 0.77, 95%CI 0.58–1.03) and IMA (HR 0.85, 95%CI 0.67–1.08), while a non-significant association was found for all-cause mortality (HR 1.03, 95%CI 0.88–1.22) and CV death (HR 1.04, 95%CI 0.85–1.28). No analysis was carried out for intracranial hemorrhage and fatal bleeding due to the small number of events.

## 4. Risk of Bias Assessment

**Figure 4:**
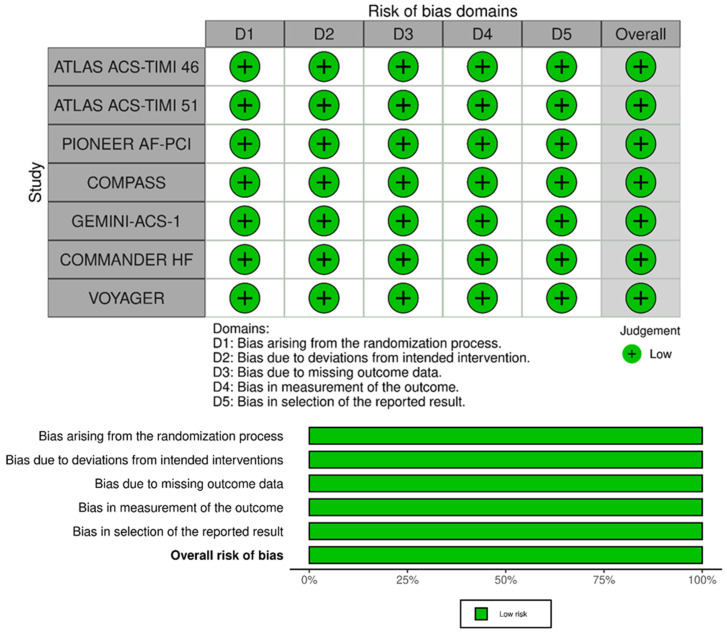
RoB2 for quality assessment of studies.

The risk of bias of RCTs included in the meta-analysis is presented in Figure 4. All studies were considered at low risk of bias for the randomization process, deviations from the intended interventions, missing outcome data, for the measurement of the outcome domain, and in the selection of reported results. Thus, all RCTs included in the meta-analysis had an overall low risk of bias.

## 5. Discussion

The main finding of our meta-analysis is that in patients with CAD and/or PAD, the use of LDR and ASA significantly reduced the risk of CVEs, and in particular, of ischemic stroke when compared to ASA alone. Second, we showed that compared to ASA treatment alone, the increased risk of major bleeding observed in patients treated with LDR and ASA was not associated with fatal bleeds or ICH. Third, the net clinical benefit between the thrombotic and hemorrhagic risk showed in our analysis supports the use of combined antithrombotic regimens containing LDR+ASA for the treatment of CAD/PAD patients with a high atherosclerotic burden. Fourth, the findings of the primary analysis were corroborated when separately assessing patients with CAD and those with PAD.

In patients who already had a first thrombotic event, despite being on top of the antithrombotic treatment with antiplatelets, persistent coagulation cascade activation may greatly contribute to the residual thrombotic risk. A recent meta-analysis showed that patients with CAD presenting with persistent clotting activation, as shown by the elevation of D-Dimer levels, were at higher risk of worse short- and long-term outcomes [33]. In addition, patients with acute MI and persistent increased prothrombin fragment levels were associated with in-hospital recurrent events and directly related to the severity of CAD defined by angiography and coronary computed tomography [34,35,36,37].

Amongst the efficacy endpoints, we found a reduced risk of stroke in patients treated with LDR, in both the overall analysis and the subgroup analyses. The importance of this finding relies on the fact that patients included in this meta-analysis were not affected by atrial fibrillation, thus suggesting that these strokes were of atherosclerotic origin. Current recommendations indicate that patients suffering from atherosclerotic stroke should be prescribed on long-term antiplatelet therapy [38]. Our data suggest that in this context LDR+ASA could be considered as a possible alternative anti thrombotic treatment and further RCTs specifically drawn to investigate these aspects in stroke patients with sinus rhythm are needed.

We should carefully consider that besides the reduction in CVEs, the association between LDR and ASA is associated with an increase in major bleeding. However, given the lack of association of LDR with fatal bleeding and ICH, we could argue that this increased risk of bleeding may be related to a higher rate of gastrointestinal bleeding in LDR treated patients, as suggested by the COMPASS study [12].

The 2023 ESC guidelines for chronic and acute coronary syndrome recommend adding LDR to ASA for long-term secondary prevention only in patients with a high-risk of ischemic events and without a high bleeding risk [8,39]. The high bleeding risk was defined as a history of intracerebral hemorrhage or ischemic stroke, recent gastrointestinal bleeding or anemia, liver failure, bleeding diathesis or coagulopathy, extreme old age or frailty, or renal failure requiring dialysis or with eGFR < 15 mL/min/1.73 m^2^. In the presence of these factors, we believe that patients requiring treatment with LDR and ASA should be carefully selected according to their thrombotic and hemorrhagic profile. The first patients who may benefit from the addition of LDR may be those with severe poly-vascular disease [40], those who already underwent an arterial coronary or peripheral revascularization, or those who suffer from lower limb amputation. In addition, the pro-active management of modifiable bleeding risk factors may help in reducing the risk of major bleeding in patients treated with LDR as a secondary prevention strategy.

### Limitations and Strengths

The analysis included only RCTs, which represent the best standard of clinical research for the assessment of the safety and efficacy of new drugs. Despite this, we acknowledge that efficacy endpoints were secondary endpoints in all RCTs. Large phase 4 observational real-world studies are needed to confirm the clinical benefit of this secondary prevention strategy. Furthermore, more than 70% of patients included in the RCTs were men, making the generalizability of our results to female patients uncertain.

## 6. Conclusions

LDR is associated with a significant reduction in recurrent CVEs and stroke in patients with CAD/PAD. This benefit is associated with an increased risk of major bleedings, which were not, however, fatal or intracranial.

## Figures and Tables

**Table 1:** Main characteristics of the studies included in the systematic review.

Author (Year)	Study Name	Setting	Age	Men (%)	FU Days	CVEs Definition	Treatment Groups	Total Number of Patients	Total Number of CVEs (%)
Mega (2009) [10]	ATLAS ACS-TIMI 46	ACS	57	76.5	210	Death, MI, stroke, or severe recurrent ischemia requiring revascularization	LDR + ASA vs. ASA	329	39 (11.9)
							LDR + DAPT vs. DAPT	976	48 (4.9)
Mega (2012) [29]	ATLAS ACS-TIMI 51	ACS	61	74.7	399	Death, MI, stroke, or severe recurrent ischemia requiring revascularization	LDR + DAPT vs. DAPT	10,227	689 (6.7)
Gibson (2016) [11]	PIONEER AF-PCI	AF with CAD	70	75.5	360	Death from cardiovascular causes, MI, or stroke	LDR + DAPT vs. warfarin + DAPT	1403	72 (5.1)
Eikelboom (2017) [30]	COMPASS	PAD with or without CAD	68	74.5	690	Death from cardiovascular causes, MI, or stroke	LDR + ASA vs. ASA	18,278	875 (4.8)
Ohman (2017) [13]	GEMINI-ACS-1	ACS	62	75	326	Cardiovascular death, MI, stroke, or definite stent thrombosis	LDR + P2Y12 vs. DAPT	3037	148 (4.9)
Zannand (2018) [14]	COMMANDER HF	HFrEF with CAD	65	77.1	633	Death from any cause, MI, or stroke	LDR + ASA with or without P2Y12 * vs. ASA	5022	1284 (25.6)
Bonaca (2020) [15]	VOYAGER	PAD	67	74	840	Acute limb ischemia, major amputation for vascular causes, MI, ischemic stroke, or death from cardiovascular causes	LDR + ASA vs. ASA	6564	1092 (16.3)

* ASA, alone or in combination with a P2Y12, was taken by 93.1% of the patients, with 34.8% taking dual antiplatelet therapy. CVEs: composite cardiovascular events, ACS: acute coronary syndrome; AF: atrial fibrillation; CAD: coronary artery disease; HFrEF: heart failure with reduced ejection fraction; ISTH: International Society on Thrombosis and Haemostasis; LDR: low-dose rivaroxaban, MI: myocardial infarction, PAD: peripheral artery disease; TIMI: thrombolysis in myocardial infarction.

**Table 2:** Definitions of major and any bleeding with the number of events for each safety endpoint.

Study Name + Treatment Arms	Number Patients LDR Group	Number Patients Control Group	Major Bleeding Deficinition	Number of Major Bleedings LDR Group, (%)	Number of Major Bleedings Control Group, (%)	Any Bleeding Definition	Number Any Bleeding LDR Group, (%)	Number Any Bleeding Control Group, (%)	Number ICH LDR Group, (%)	Number ICH Control Group, (%)	Number Fatal Bleedings LDR Group, (%)	Number Fatal Bleeding Control Group, (%)
ATLAS ACS-TIMI 46 LDR + ASA vs. ASA	77	252	TIMI major bleeding	-	-	Clinically significant bleeding (TIMI major, TIMI minor, or requiring medical attention)	1 (1.3)	4 (1.6)	-	-	-	-
ATLAS ACS-TIMI 46 LDR + DAPT vs. DAPT	75	901					6 (8)	33 (3.7)				
ATLAS ACS-TIMI 51 LDR + DAPT vs. DAPT	5114	5113	TIMI major bleeding not associated with CABG	65 (1.3)	19 (0.4)	TIMI bleeding requiring medical attention	492 (9.6)	282 (5.5)	14 (0.3)	5 (0.1)	6 (0.1)	6 (0.1)
PIONEER AF-PCI LDR + DAPT vs. warfarin + DAPT	706	697	TIMI major bleeding	12 (1.7)	20 (2.9)	TIMI clinically significant bleeding	117 (16.6)	167 (24.0)	-		-	-
COMPASS LDR + ASA vs. ASA	9152	9126	ISTH Major bleeding modified ^§^	206 (2.3)	116 (1.3)	Calculated adding minor and major TIMI bleedings	1044 (11.4)	673 (7.4)	28 (0.3)	24 (0.3)	15 (0.2)	10 (0.1)
GEMINI-ACS-1 LDR + P2Y12 vs. DAPT	1519	1518	TIMI major bleeding	10 (0.7)	8 (0.5)	TIMI non-CABG clinically significant bleeding	80 (5.3)	74 (4.9)	1 (0)	0 (0)	0 (0)	2 (0)
COMMANDER HF LDR + ASA vs. ASA	2507	2515	ISTH major bleeding	82 (3.3)	50 (2.0)	Bleeding requiring hospitalization	61 (2.4)	48 (1.9)	-	-	9 (0.4)	9 (0.4)
VOYAGER LDR + ASA vs. ASA	3286	3278	TIMI major bleeding	62 (1.9)	44 (1.3)	N/A	-	-	13 (0.4)	27 (0.8)	6 (0.2)	6 (0.2)

ASA: aspirin, DAPT: dual antiplatelet therapy, LDR: low-dose rivaroxaban, ICH: intracranial hemorrhage, TIMI: thrombolysis in myocardial infarction. ^§^: included fatal bleeding, symptomatic bleeding into a critical organ, bleeding into a surgical site requiring reoperation, and bleeding that led to hospitalization.

**Table 3:** Number of events for each efficacy endpoint.

Study Name + Treatment Arms	Number Patients LDR Group (%)	Number Patients Control Group (%)	Number of CVEs LDR Group (%)	Number of CVEs Control Group (%)	Number MI LDR Group (%)	Number MI Control Group	Number Stroke LDR Group	Number Stroke Control Group	Number All-Cause Mortality LDR Group	Number All-Cause Mortality Control Group	Number CV Death LDR Group	Number CV Death Control Group
ATLAS ACS-TIMI 46 LDR + ASA vs. ASA	77	252	-	-	-	-	-	-	-	-	-	-
ATLAS ACS-TIMI 46 LDR + DAPT vs. DAPT	75	901	-	-	-	-	-	-	-	-	-	-
ATLAS ACS-TIMI 51 LDR + DAPT vs. DAPT	5114	5113	65 (1.3)	19 (0.4)	205 (4.0)	229 (4.5)	46 (0.9)	41 (08)	103 (2.0)	153 (3.0)	94 (1.8)	143 (2.8)
PIONEER AF-PCI LDR+DAPT vs. warfarin + DAPT	706	697	12 (1.7)	20 (0.4)	17 (2.4)	21 (3.0)	10 (1.4)	7 (1.0)	-	-	14 (2.0)	11 (1.6)
COMPASS LDR + ASA vs. ASA	9152	9126	206 (2.3)	116 (1.3)	178 (1.9)	205 (2.2)	83 (0.9)	142 (1.6)	313 (3.4)	378 (4.1)	160 (1.7)	203 (2.2)
GEMINI-ACS-1 LDR + P2Y12 vs. DAPT	1519	1518	10 (0.7)	8 (0.5)	56 (3.7)	49 (3.2)	7 (0.5)	12 (0.8)	22 (1.4)	23 (1.5)	19 (1.3)	17 (1.1)
COMMANDER HF LDR + ASA vs. ASA	2507	2515	82 (3.3)	50 (2.0)	98 (3.9)	118 (4.7)	51 (2.0)	76 (3.0)	546 (21.8)	556 (22.1)	453 (18.1)	476 (18.9)
VOYAGER LDR + ASA vs. ASA	3286	3278	62 (1.9)	44 (1.3)	131 (4.0)	148 (4.5)	71 (2.2)	82 (2.5)	321 (9.8)	297 (9.1)	199 (6.1)	174 (5.3)

ASA: aspirin, CVEs: cardiovascular events; MI: myocardial infarction, LDR: low-dose rivaroxaban, DAPT: dual antiplatelet therapies.

## Data Availability

The data utilized for this study has been extracted from the cited RCTs and reported in the manuscript.

## Supplementary Materials

The following supporting information can be downloaded at: https://www.mdpi.com/article/10.3390/jcm13072033/s1, Figure S1: Funnel plots for safety endpoints; Figure S2: Funnel plots for efficacy endpoints; Figure S3: Efficacy endpoints in patients with coronary artery disease; Figure S4: Safety endpoints in patients with coronary artery disease; Figure S5: Safety and efficacy endpoints in patients with peripheral artery disease; Table S1: Literature online search strategies.
