# Supplementary material for: Efficacy and Safety of Combination Therapy with Low-Dose Rivaroxaban in Patients with Cardiovascular Disease: A Systematic Review and Meta-Analysis of Randomized Controlled Trials
Source: J Clin Med. 2024 Mar 31;13(7):2033. doi: 10.3390/jcm13072033 (PMC11012887; doi:10.3390/jcm13072033)

Efficacy and safety of combination therapy with low dose rivaroxaban in patients with cardiovascular disease: a systematic review and meta-analysis of randomized controlled trials

Tommaso Bucci, Francesco Del Sole, Danilo Menichelli, Gioacchino Galardo, Flavio Giuseppe Biccirè, Alessio Farcomeni, Gregory Y.H. Lip, Pasquale Pignatelli, Daniele Pastori

Supplementary materials

Supplementary Table S1. Literature online search strategies.

Database	Search strategy
PubMed	‘rivaroxaban’ AND ‘coronary artery disease’ OR ‘peripheral artery disease’
Embase	' rivaroxaban' /exp AND ‘coronary artery disease’ OR ‘peripheral artery disease’ /exp
Web of Science	ALL= rivaroxaban ' AND ALL= ‘coronary artery disease’ OR ‘peripheral artery disease’

Supplementary Figure S1. Funnel plots for safety endpoints.

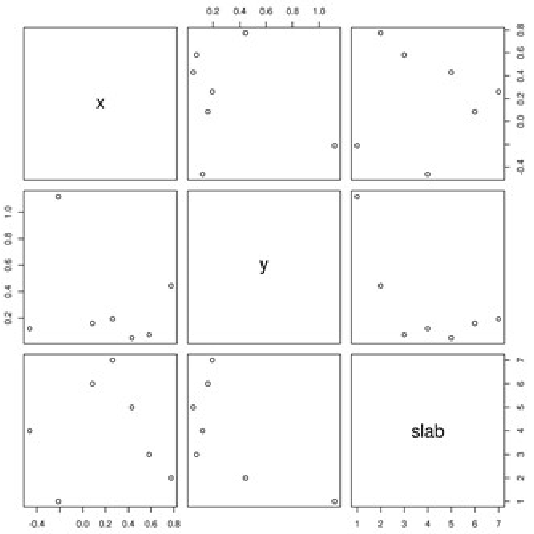

Any bleeding

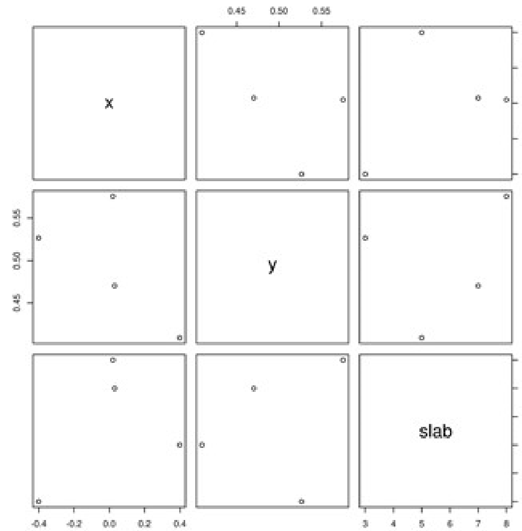

Fatal bleeding

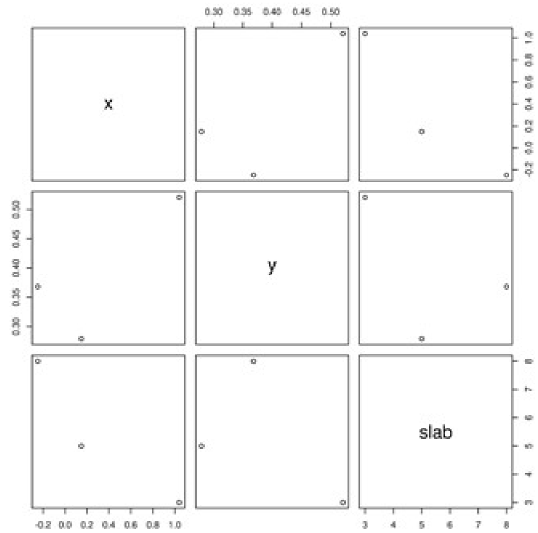

ICH

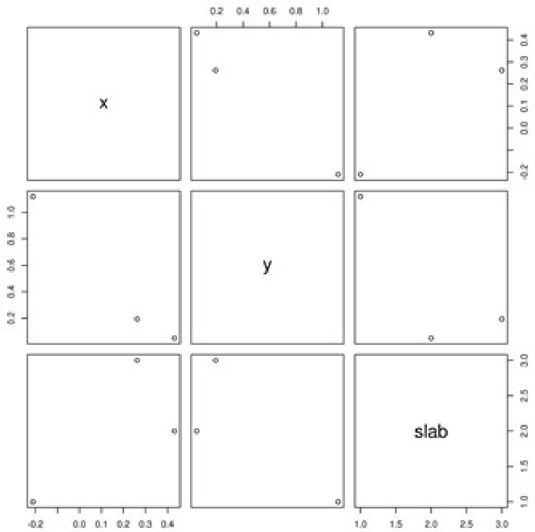

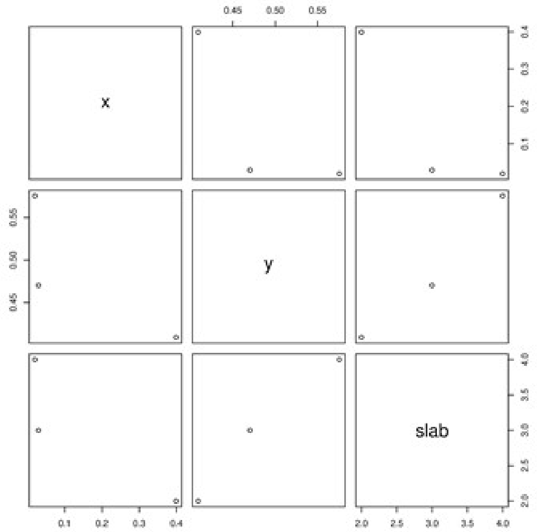

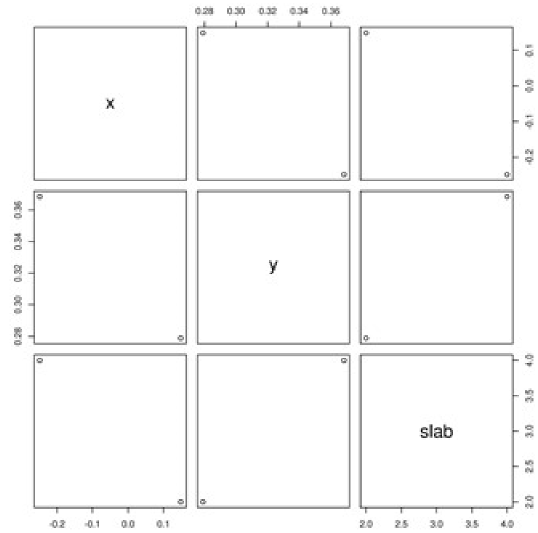

Supplementary Figure S2. Funnel plots for efficacy endpoints.

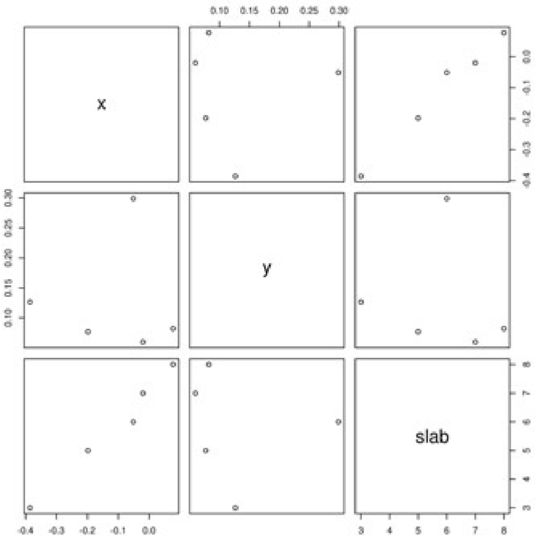

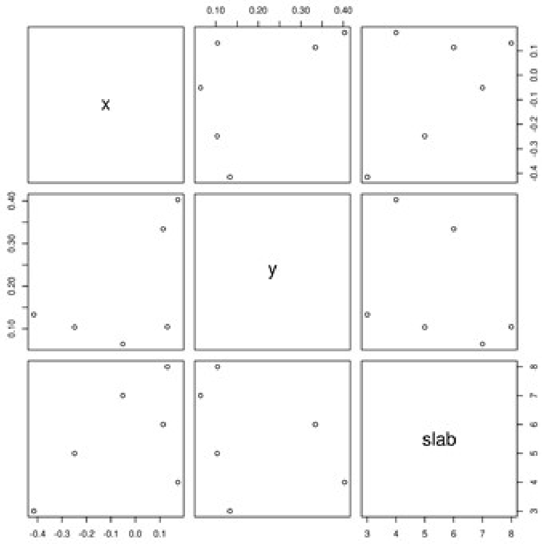

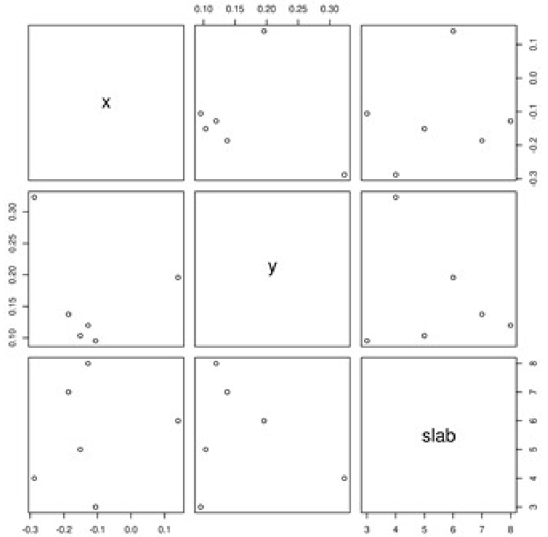

All cause mortality

CV mortality

Myocardial Infarction

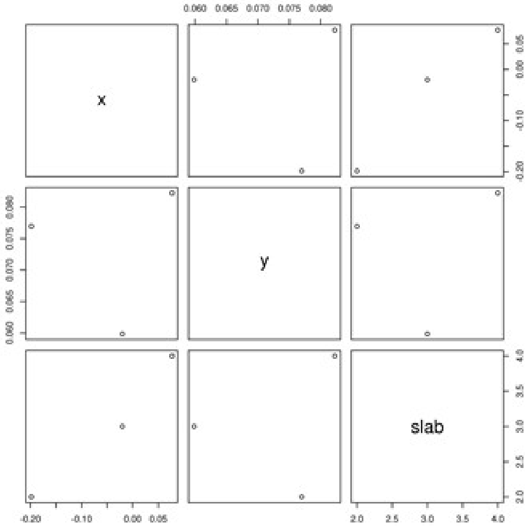

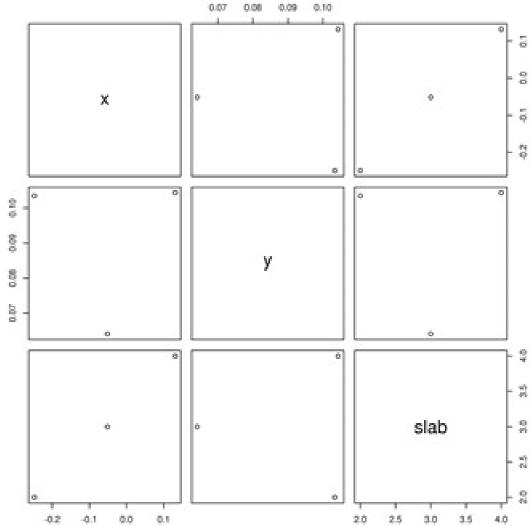

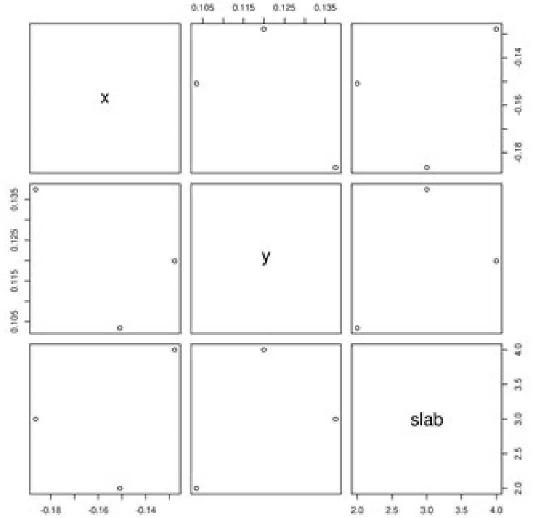

Supplementary Figure S3. Efficacy endpoints in patients with coronary artery disease.

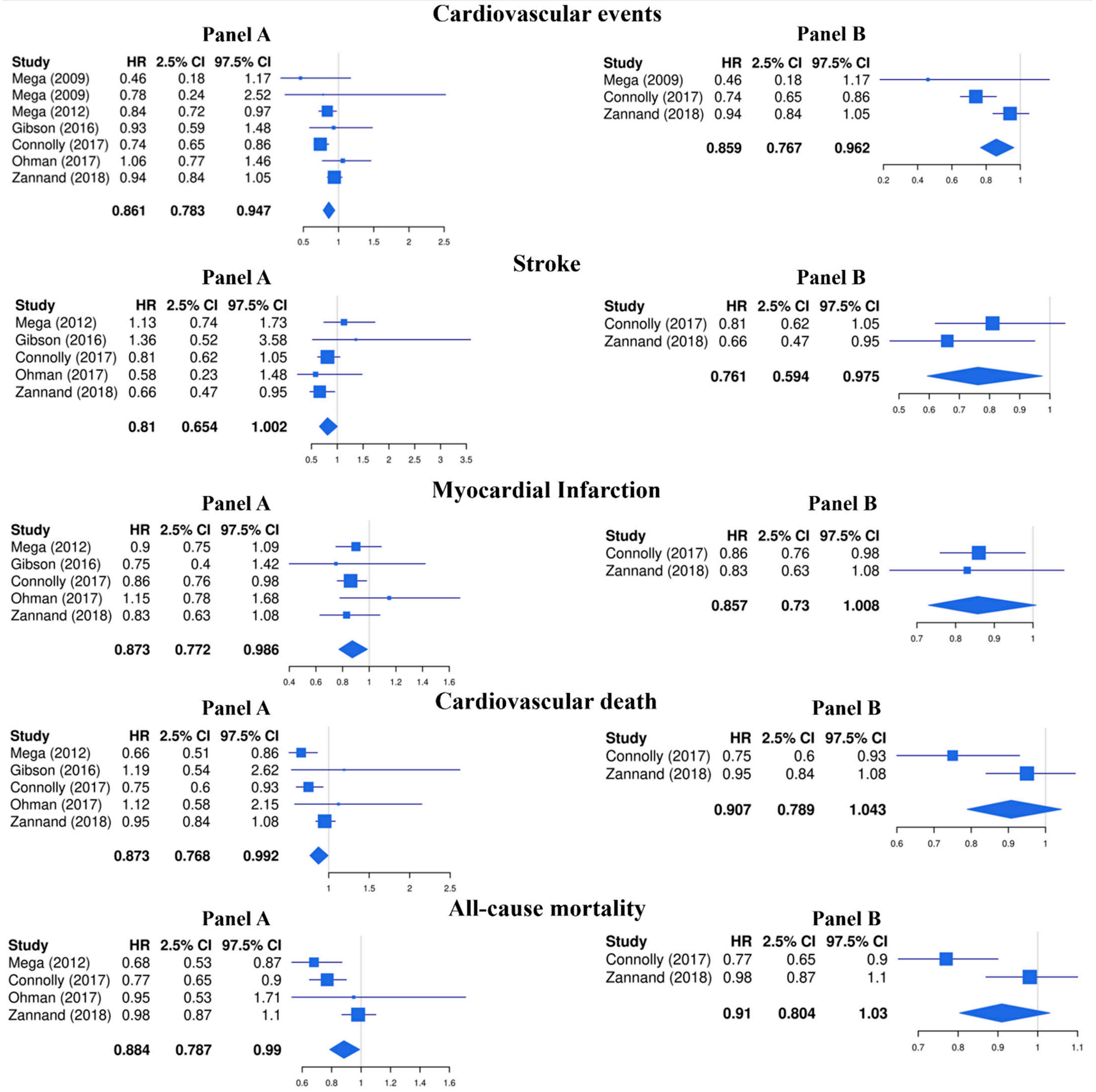

Supplementary Figure S4. Safety endpoints in patients with coronary artery disease.

Major bleeding

Panel A

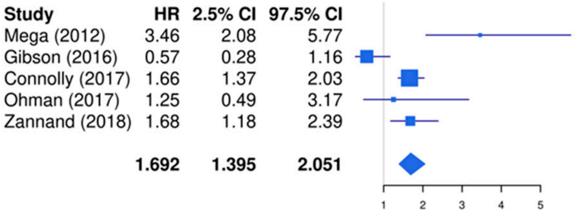

Panel B

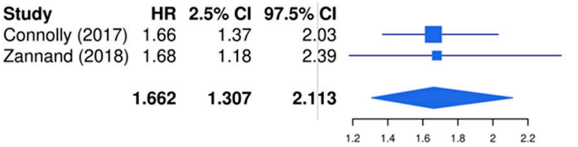

Any bleeding

Panel A

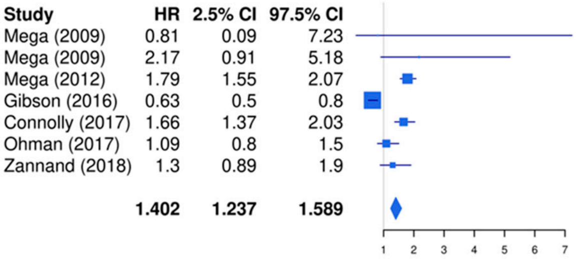

Panel B

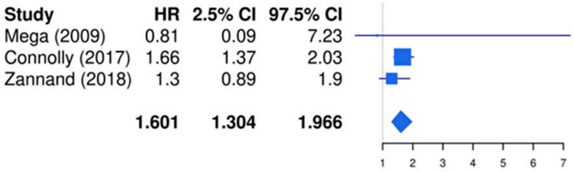

Intracranial haemorrhage

Panel A

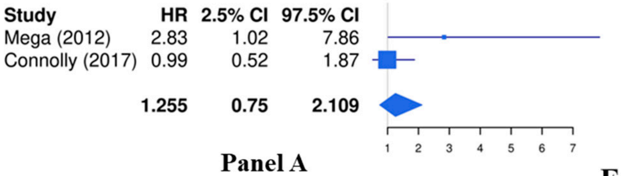

Fatal beeding

Panel A

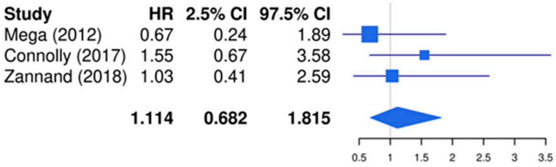

Supplementary Figure S5. Safety and efficacy endpoints in patients with peripheral artery disease.

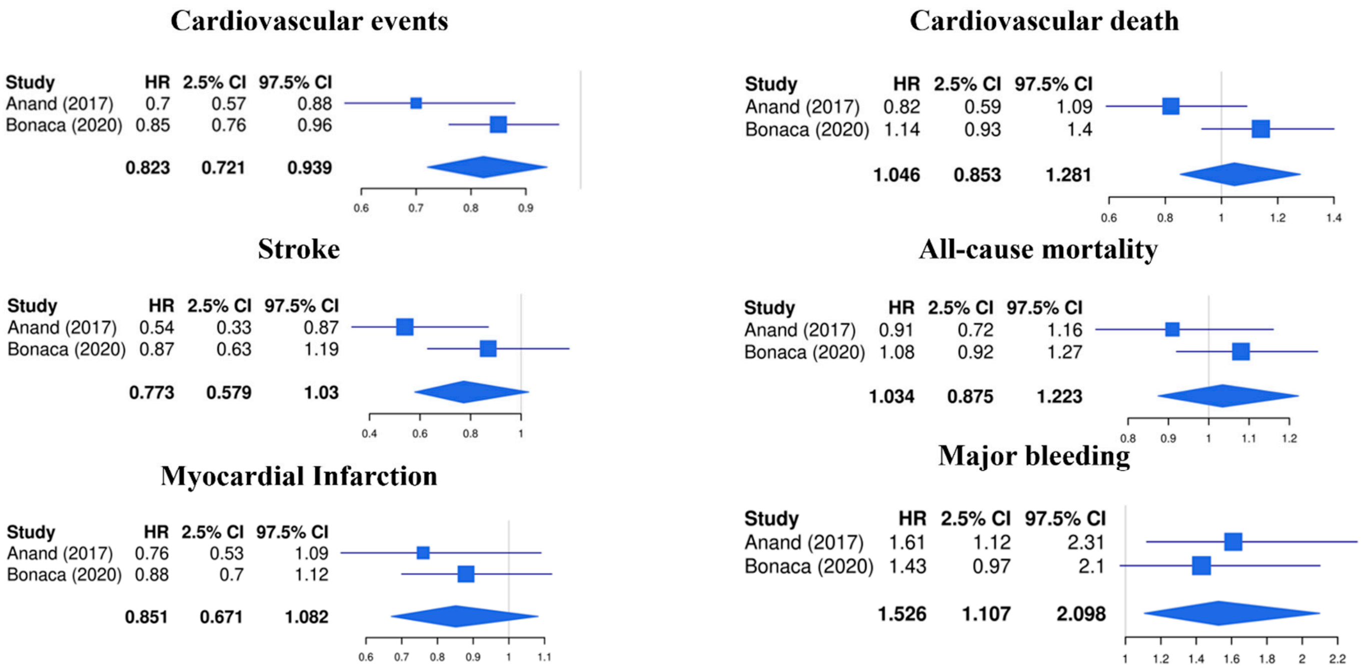

Supplement: Supplementary file 1 [file jcm-13-02033-s001.zip › jcm-2924988-supplementary.pdf]
